# Posterior pituitary tumors and other rare entities involving the pituitary gland

**DOI:** 10.1111/bpa.13307

**Published:** 2024-09-30

**Authors:** Federico Roncaroli, Caterina Giannini

**Affiliations:** ^1^ Geoffrey Jefferson Brain Research Centre, Division of Neuroscience, Faculty of Biology, Medicine and Health University of Manchester Manchester UK; ^2^ Division of Anatomic Pathology Mayo Clinic College of Medicine Rochester Minnesota USA; ^3^ Department of Biomedical and Neuromotor Sciences (DIBINEM) Alma Mater Studiorum University of Bologna Bologna Italy

**Keywords:** primary papillary epithelial tumor of the sella, atypical teratoid rhabdoid tumor, pituicytoma, pituitary, sella, thyroid transcription factor 1

## Abstract

Non‐neuroendocrine tumors account for around 10% of all primary neoplasms of the sella. If meningiomas, craniopharyngiomas, and germ cell tumors are excluded, the remaining lesions include a broad spectrum of uncommon, benign, and aggressive, often diagnostically challenging lesions. This review aims to summarize the essential clinicopathological features of tumors of the posterior pituitary gland, infundibulum spectrum expressing thyroid transcription factor 1, and primary sellar atypical rhabdoid teratoid tumor, and provide the criteria for their diagnosis and management.

## 1 INTRODUCTION

Primary non‐neuroendocrine tumors of the adeno and neurohypophysis account for about 10% of sellar neoplasms [1]. If craniopharyngiomas, germinomas, and meningiomas are excluded, the remaining tumor entities include a spectrum of rare neoplasms presenting with signs and symptoms of mass effect and less commonly with endocrine dysfunction.

This review will focus on the recent advances in the classification, and nomenclature of tumors of the posterior pituitary and infundibulum (TPPIs), and it will provide a practical summary of the clinical, neuroimaging, pathological, and molecular features of primary sellar atypical teratoid rhabdoid tumor (sAT/RT) as well as the recently described primary papillary epithelial tumor of the sella (PPETS), both of which are not described in the fifth edition of the World Health Organisation (WHO) Classification of Endocrine Tumors (ENDO5).

## 2 THYROID TRANSCRIPTION FACTOR 1 EXPRESSING TUMORS OF THE POSTERIOR HYPOPHYSIS AND INFUNDIBULUM

**FIGURE 1:**
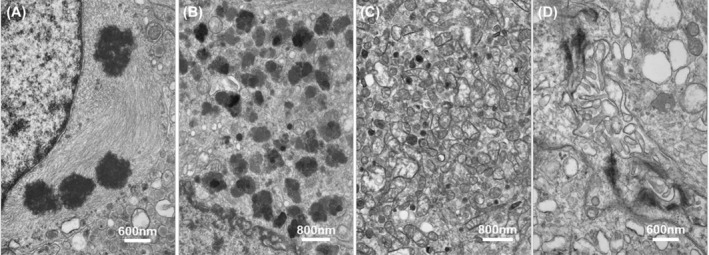
Ultrastructural features of normal human pituicytes include: major type showing an electron‐lucent matrix of fibrils that contains organelles (A); dark type characterized by accumulation of electron‐dense lysosomes (B); oncocytic with the perikaryon filled with mitochondria (C); ependymal characterized by cell‐to‐cell junctions and lumina lined by microvilli (D).

Tumors of the posterior hypophysis and infundibulum represent a family of neoplasms characterized by the expression of thyroid transcription factor‐1 (TTF‐1) [2]. The group comprises pituicytoma (PTC), granular cell tumor of the Sellar Region (GCT), spindle cell oncocytoma (SCO) the so‐called sellar ependymoma (SE), tumors with hybrid features, and PPET. Lee et al. first demonstrated TTF‐1 expression in TPPIs [3], observed similarities between TPPIs and major, dark, granular, oncocytic, and ependymal pituicytes as described by Takei et al. [4] (Figure 1), and proposed TPPIs may represent the neoplastic counterpart of normal pituicytes. Such observations were later confirmed by Mete et al. [5], eventually leading to the position in ENDO5 that TPPIs are variants of PTC.

## 3 NOMENCLATURE

The discussion on the nomenclature of TPPIs remains open. The chapter in the 5th Edition of the WHO Classification of Tumors of the Central Nervous System (CNS5) [2] acknowledged they represent a spectrum of neoplasms likely originating from pituicytes, but opted to maintain SCO, PTC, and GCTs as distinct lesions with the aim to emphasize their microscopic features, spectrum of immunophenotypic and molecular profiles, as well as the differences in their behavior. SE was not included in CNS5 because of its rarity [2]. In contrast, ENDO5 [6] supported a unified definition for these tumors as a single entity, with SCO, GCT, and SE being considered morphological variants of PTC. It is of note, however, that the introductory chapter on pituitary tumors in ENDO5 is less prescriptive and still recognizes that patient demographics and clinical outcomes vary within the TPPI spectrum, “*warranting their recognition as distinct subtypes*” [7]. Irrespective of their classification, TPPIs are regarded as WHO grade 1.

Considering the uncertainties in the classification and the absence of a unifying driver mutation or proven common pathogenesis, a diagnosis of “*TTF‐1 expressing tumor of the posterior pituitary and infundibulum with features of PTC, SCO, GCT, or SE*” seems more appropriate, particularly if implemented alongside the stratification into three clinically relevant types proposed by Schmid et al. [8], with GCT being a separate group and SCO and PTC being further stratified by presence or absence of copy number variation (CNV).

In this review, we have retained the terms PTC, SCO, GCT, and SE rather than PTC with oncocytic, granular cell, or ependymal features.

## 4 THYROID TRANSCRIPTION FACTOR 1

TTF‐1 expression is the hallmark of TPPIs and defines their common origin [3] but its role in their pathogenesis has not been demonstrated.

TTF‐1 or thyroid‐specific enhancer binding protein, is a phylogenetically conserved, developmentally tightly regulated nuclear transcription factor of 371 amino acids, encoded by the NKX2.1 homeobox gene mapped in humans on chromosome 14q13 [9].

NKX2.1 drives normal lung and thyroid development, as well as the ventral forebrain, including the median eminence and the preoptic area, ependymal and subependymal cells of the third ventricle, the infundibulum, and the posterior pituitary. Its relevance to the homeostasis of the adult neurohypophysis has only been partly investigated [9]. Although NKX2.1 protein and mRNA are absent in the anterior or intermediate pituitary lobes during development, the anterior gland does not develop in NKX2.1 double knock‐out animals [9, 10].

NKX2.1 heterozygous mutations usually occur de novo and follow an autosomal dominant inheritance. In humans, they cause the brain–lung–thyroid syndrome, now defined as NKX2.1‐related disorders [11] that usually manifest in the first year of life. Most mutations in NKX2.1 gene result in haploinsufficiency, with some mutations resulting in the generation of a protein with a dominant‐negative effect on the wild‐type protein [10, 11]. No TPPIs have been described in patients with NKX2.1 mutations.

NKX2.1 is well known to play a role in the pathogenesis of thyroid follicular, papillary, and medullary carcinoma, as well as lung non‐mucinous adenocarcinoma and neuroendocrine carcinoma, but its role in the pathogenesis of TPPIs has not been explored. NKX2.1 can have both oncogenic and tumor suppressor functions, which likely depend on the cellular context [10].

Pathologists should be aware that the three routinely used TTF‐1 antibody clones 8G7G3/1, SPT24, and SP141 have different sensitivity and specificity and can therefore result in variable distribution and intensity of immunohistochemical stain [12], or even in nonspecific staining of other tumor types [13, 14]. The 8G7G3/1 clone is directed against the 393‐amino acid recombinant rat TTF‐1 protein, while the 123 amino acid sequence of the N‐terminus of human protein was used as an immunogen for SPT24. The rabbit monoclonal antibody SP141 has reportedly been raised against the human recombinant protein. Lee et al. observed weaker staining in PTC with 8G3G3/1 than SPT24 and reported that the performance of these clones may be affected by decalcification and prolonged formalin fixation [3].

## 5 EPIDEMIOLOGICAL, CLINICAL, AND IMAGING FEATURES

Data from the National Cancer Database report an incidence for TPPIs of around 0.2% of all sellar tumors in the adult population, with the caveat that figures are likely underestimated due to inaccurate International classification of diseases for oncology (ICD‐O) coding [15]. PTC is the most common TPPIs, accounting for 3.27% of sellar neoplasms [1]. The reported annual incidence in the Surveillance, Epidemiology, and End Results database is 0.017% for SCO, 0.023% for GCT [15]. The figures are lower in children [16, 17]. The incidence of SE is unknown given its rarity. TPPIs occur in adults with a peak of age between 50 and 60 years. Patients affected by SCO are slightly older, with a mean age of 61.6 years; no striking male or female predominance has been reported [1, 17, 18].

Headache and visual field defects, hypopituitarism or increased prolactin, secondary to stalk compression are the most common symptoms and signs at onset [17], with possible suppression of the hypothalamic/pituitary axes. In rare instances, PTC and GCT cause acromegaly or Cushing's disease in the absence of a synchronous adenohypophyseal tumor, but the mechanism is unclear [19, 20, 21, 22]. In contrast, growth hormone excess and hypercortisolism have never been documented in patients with SCO [20]. Despite their origin from the posterior pituitary and infundibulum, only 3%–5% of patients present with arginine vasopressin deficiency (AVD) [23], formerly known as diabetes insipidus [24]. Unlike PTC and GCT, SCO is prone to spontaneous or intraoperative hemorrhage, at times causing serious complications [18, 25, 26, 27, 28].

Pre‐ and post‐contrast structural imaging of TPPIs shows overlapping features with pituitary neuroendocrine tumors (PitNET)/adenomas, including isointensity on T1‐weighted and heterogeneous intensity of T2‐sequencies [29, 30]; TPPIs may show however bright homogeneous or heterogeneous enhancement following gadolinium administration [31, 32]. Cystic changes are uncommon. Features distinctive of SCO include multiple hypointense foci and linear voids on the T1‐weighted sequences due to frequent hemosiderin deposits and prominent vascularity [31, 33]. Notably, the anterior hypophysis is typically involved in SCO and therefore not visible on pre‐operative scans, while PTC and GCT are more often suprasellar [29, 31]. Extension to the cavernous sinus, invasion of the sellar floor [27] with invasion of the sphenoid sinus have been seen in SCO [23].

Surgery is the treatment of election for TPPIs, and gross total resection is considered curative. While the rate of total removal is lower than PitNET/adenomas, many patients with residual TPPI remain stable and do not require further intervention [31, 34]. Firm consistency and marked vascularity, as well as poor dissection planes from the surrounding structures, impact surgery [27, 35]. Intraoperative embolization can be necessary in cases of SCO with uncontrollable bleed [36]. Common post‐operative complications include hypopituitarism and transient or permanent AVD [17].

Cases of TPPIs with aggressive behavior have been documented [17]. Outcomes vary depending on tumor type, with SCO showing the greatest propensity to recur compared to the other TPPIs [37, 38, 39, 40, 41]. A recent, comprehensive literature review on SCO and GCT found that 27% of SCOs had recurrence or progression, while only 5% of GCTs progressed or recurred [15, 42]. No accepted pathological criteria exist to identify, high‐risk, potentially aggressive TPPIs.

The efficacy of postoperative radiotherapy including stereotactic radiosurgery is still under debate. Over 60% of incompletely resected SCO were treated stereotactic radiosurgery or standard fractionated radiotherapy [15]. Data from the literature on disease control following radiation is still inconclusive although a marginal improvement of recurrence rate has been suggested [27, 34]. Earlier studies indicated that 72% of SCOs recurred after adjuvant stereotactic radiosurgery or fractionated radiotherapy, while more recent studies suggest a better control of the residual disease [27].

## 6 LIGHT MICROSCOPIC FEATURES, IMMUNOPROFILE, AND ULTRASTRUCTURE

Pathological features distinctive of each TPPI are well described. They share TTF‐1 nuclear expression and the absence of endocrine markers such as chromogranin‐A and Insulinoma‐associated protein 1, pituitary hormones, and lineage restricted pituitary transcription factors.

**FIGURE 2:**
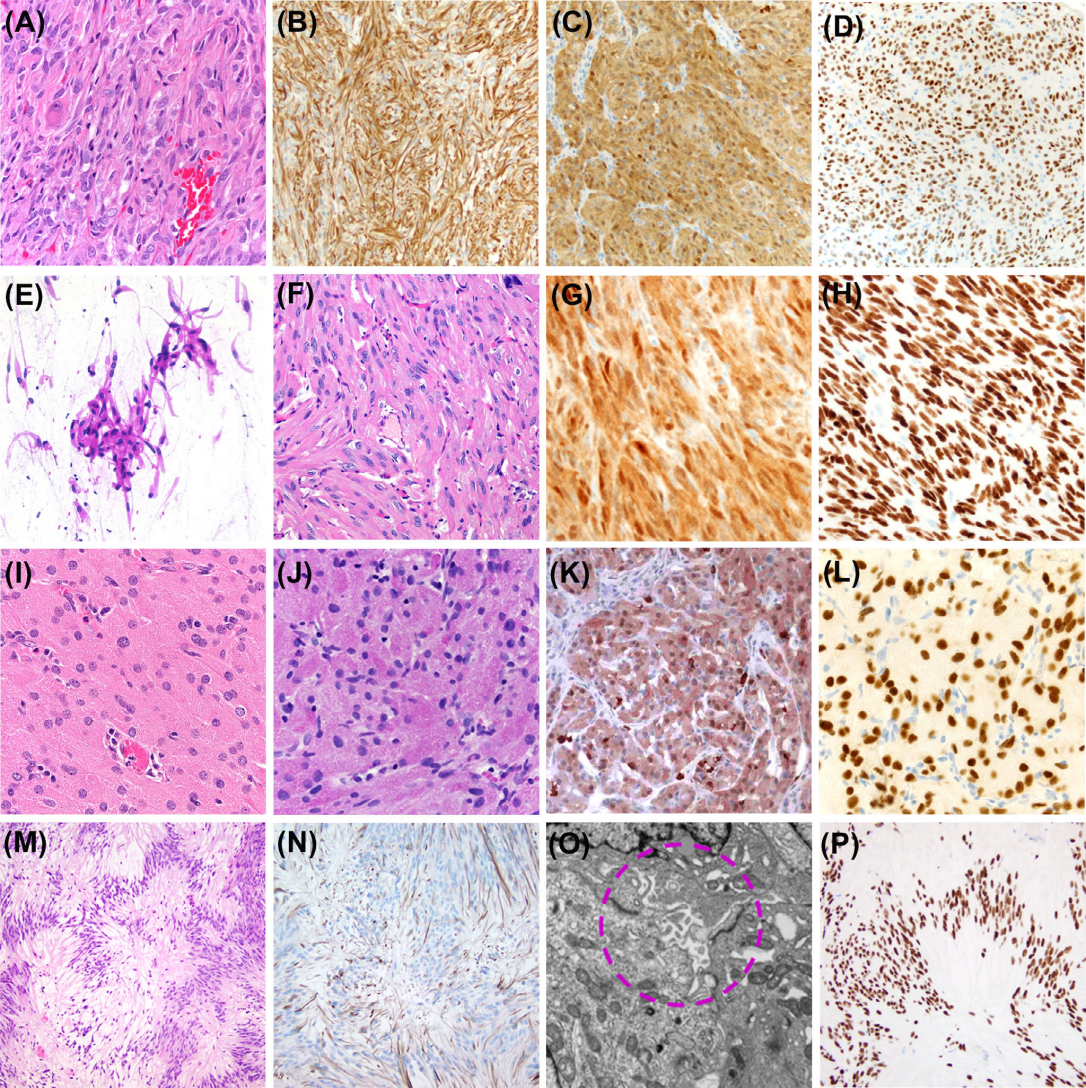
This panel shows the essential light microscopic features of posterior tumors of the pituitary and infundibulum, Pituicytoma, shows fascicles of bipolar cells (A—hematoxylin and eosin [H&E], ×20) expressing Glial fibrillary acidic protein (GFAP) (B—immunoperoxidase, ×20), S100 (C—immunoperoxidase, ×20), and thyroid transcription factor‐1 (TTF‐1) (D—immunoperoxidase, ×20). Spindle cell oncocytoma is characterized by spindle cells with eosinophilic, granular cytoplasm (E—intraoperative smear, H&E, ×20; F—H&E, ×20) that show widespread staining for S100 protein (G—immunoperoxidase, ×20) and TTF‐1 (H—immunoperoxidase, ×20). Sheets of large cells with granular, intensely eosinophilic cytoplasm characterize granular cell tumor (J—H&E, ×20); the cells display intense periodic acid–Schiff (PAS) staining (K—PAS, ×20) and expression of S100 protein (L—immunoperoxidase, ×20) and TTF‐1 (M—immunoperoxidase, ×20). This case of sellar ependymoma shows spindle cells with fibrillary cytoplasm forming fascicles and perivascular pseudo‐rosettes; there is nuclear clustering (M—H&E, ×20); a few cells express GFAP (N—immunoperoxidase, ×20) while they show widespread, intense TTF‐1 expression (P—immunoperoxidase, ×20); electron microscopy demonstrates extracellular lumina lined with microvilli (circle) (O).

The typical pathological features of PTC, SCO, GCT, and SE are represented in Figure 2.

## 7 PITUICYTOMA

PTCs were first described in 1955 [43] but the diagnostic criteria were only defined by Brat et al. [44]; the WHO recognized them as a separate entity in the third edition in 2007. Its hallmark microscopic features include compact fascicles or sheets of spindle cells with bipolar fibrillary cytoplasm and a mildly atypical nucleus. A storiform pattern is common. Focal lymphocytic infiltrates are seen at times. No Rosenthal's fibers, eosinophilic granular bodies, myxoid changes, and calcification are seen [5, 44]. Epithelioid cells can also be a rare feature [45].

Mitotic activity and Ki‐67 labeling index (LI) are low [17, 18]. However, a few PTCs can display atypical cells, increased cellularity, mitotic activity, and a Ki‐67 LI higher than 5% [46, 47], but their prognostic relevance is unclear. PTCs stain for vimentin and S100 protein; with variable Glial fibrillary acidic protein (GFAP) expression. A minority of cases express Epithelial membrane antigen (EMA), CD56, galectin 3, CD68, and Bcl‐2, which is usually focal [5]. There can be focal synaptophysin expression [47]. Olig2 staining has been reported [5, 48]. Cytokeratins and neurofilaments have not been described. The distinction between PTC and normal posterior pituitary in small biopsies can be challenging; however, the presence of axons crossing the biopsy and diffuse expression of vasopressin favor normal tissue. Differential diagnosis based solely on the identification of Herring bodies can be misleading as they have been identified in rare examples of PTC [49, 50, 51, 52]. Correlation with neuroimaging is essential.

## 8 SPINDLE CELL ONCOCYTOMA

**FIGURE 3:**
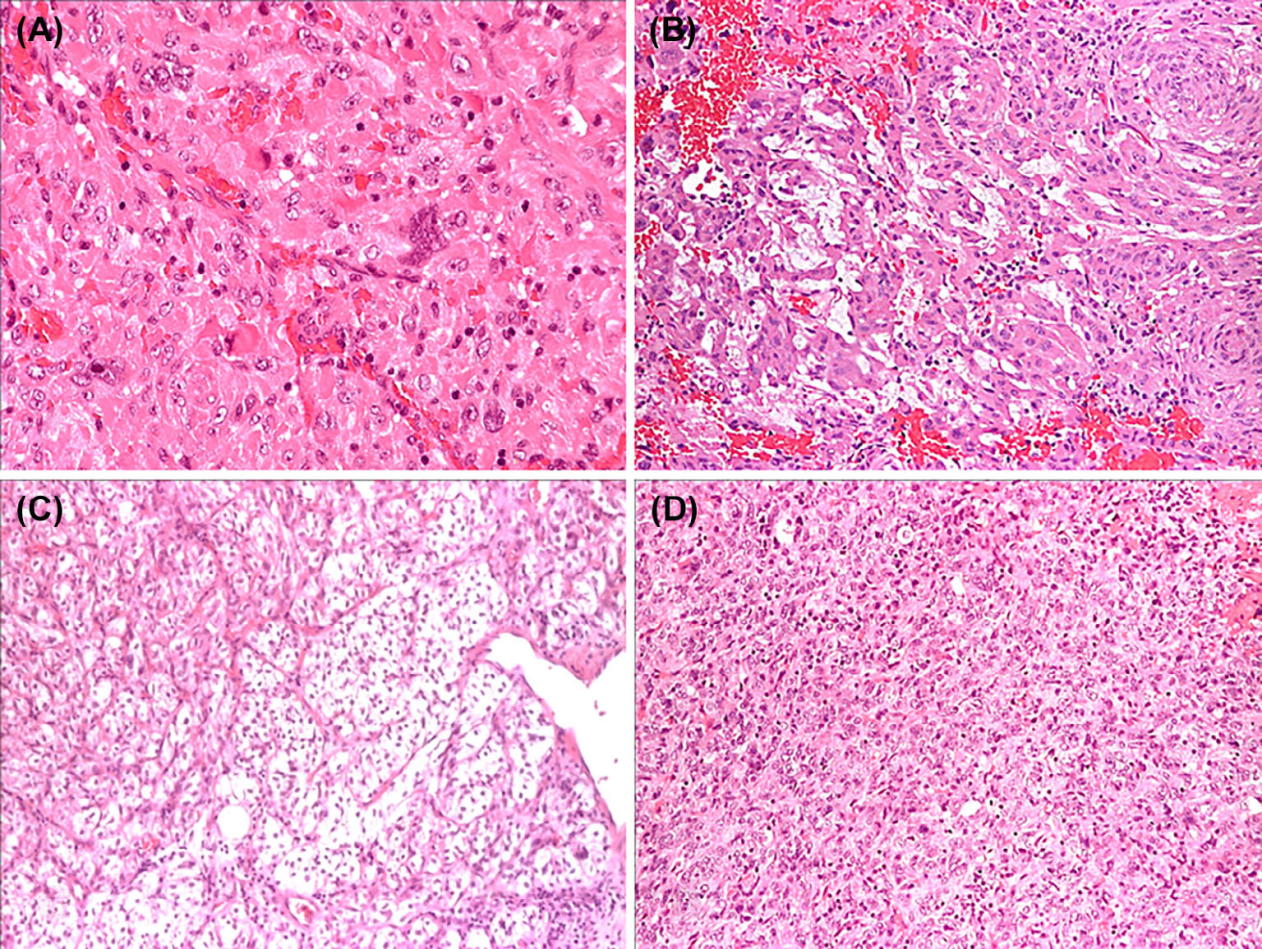
This panel represents the spectrum of light microscopic features seen in spindle cells oncocytoma including tumor cells with large, bizarre nucleus (A—hematoxylin and eosin [H&E], ×20), focal stromal myxoid changes (B—H&E, ×20), areas composed of clear cells (C—H&E, ×20), and sheets of small cells with scanty cytoplasm (D—H&E, ×20).

The term SCO was coined in 2002 [53] to describe a primary sellar and suprasellar tumor characterized by fascicles, sheets, or lobules composed of spindle or epithelioid cells with granular, eosinophilic cytoplasm due to an excess of mitochondria. In the original study, the definition of SCO was limited to those lesions expressing EMA and S100 protein while lacking GFAP to separate them from PTC. Cell‐to‐cell junctions were also regarded as a distinguishing feature. The several reports that followed more accurately defined the spectrum of this lesion. Oncocytic change can be focal; unusual features include whorls, cells with vacuolated cytoplasm, osteoclast‐like cells, myxoid stromal changes, and nuclear pleomorphism (Figure 3). Several cases show lymphocytic infiltrates, which may aggregate in follicles. The immunoprofile of SCO includes vimentin, S‐100 protein, diffuse or focal membranous EMA, annexin 1, galectin‐3, and somatostatin receptors [54]. The anti‐mitochondria antibody highlights oncocytic cells. Other markers less commonly described and always in a minority of cells include GFAP [5, 55], synaptophysin [38], Neural cell adhesion molecule (CD56), alpha‐B‐crystallin, Bcl‐2, CD44, nestin, cytokeratin, extracellular signal‐regulated kinase (ERK), AKT, S6 proteins, and D2 dopamine receptors [54]. Where reported, mitoses are usually negligible and the Ki‐67 LI low. However, a few examples showing noticeable mitotic activity and high Ki‐67 LI have been reported [35], with a suggestion that a Ki‐67 LI higher than 5% may predict a high risk of recurrence [27, 56]. Ultrastructurally, the cytoplasm of tumor cells often appears packed with abnormal mitochondria. Desmosomes and intermediate‐type junction are typically seen, unlike in the other TPPIs [57, 58]. Sparse, small neurosecretory granules are also a feature of SCO [5, 38, 59]. One case contained premelanosomes and it was initially diagnosed as amelanotic melanocytoma [60].

## 9 GRANULAR CELL TUMOR

Light microscopic features of sellar GCT are similar to GCTs from any other site. Its diagnosis is therefore straightforward, with the lesion showing the typical densely cellular sheets, fascicles, or lobules surrounded by thin‐walled vessels and composed of polygonal cells with eosinophilic, granular, periodic acid–Schiff‐positive, and diastase‐resistant cytoplasm. Lymphocyte and macrophage infiltrates can be seen. Mitoses are rare; necrosis is rarely seen. Intense and widespread expression of vimentin, S100 protein, CD68, alpha‐1‐antitrypsin, and cathepsin B is present throughout [5, 61]. Single cases of atypical GCTs characterized by nuclear atypia and mitotic activity have been described [62, 63]. At ultrastructural examination, the cytoplasm appears packed with lysosomes while secretory granules are absent.

## 10 SELLAR EPENDYMOMA

This very uncommon tumor was already regarded as a variant of PTC in its original description [64]. Histologically, SE show sheets and small fascicles of spindled to epithelioid cells, along with perivascular pseudo‐rosettes, true ependymal rosettes, and follicle‐like structures reminiscent of ependymoma. GFAP, vimentin, and S100 protein are variably expressed. EMA shows membranous, luminal, and para‐nuclear dot‐like pattern [64, 65, 66, 67]. Limited staining for CAM 5.2, CD99, Bcl‐2, and Galectin‐3 has been documented [64, 68]. Intra‐ and inter‐cellular lumina lined by microvilli and cilia, intermediate filaments, microtubules, and the presence of junctions have been described at electron microscopy [64, 66].

## 11 UNUSUAL MICROSCOPIC PATTERNS AND MIXED TUMORS

Rare TPPIs display combined features of SCO, PTC, and SE [49, 68] or a follicular pattern.

Vajtai et al. described a TPPI with follicle‐like structures and ultrastructural features of epithelial differentiation and microvilli. Areas of transition from the fascicular pattern to follicles were present, including poorly cohesive tumor cells, slit‐like channels, or nests with a central space. EMA was expressed on the surface of follicle‐like structures [69]. Yoshimoto et al. [70] reported another example of hybrid features of PTC and SCO: equivocal ependymal rosettes, follicle‐like structures, and cell nests with microlumens. The lesion lacked GFAP and S100 protein, showed apical EMA expression in follicle‐like structures and intracytoplasmic lumina, and exhibited cytokeratin CAM5.2 and neurofilament staining in a few cells, alongside weak, possibly nonspecific synaptophysin and chromogranin. Electron microscopy demonstrated accumulation of mitochondria, intermediate junctions, and microvilli in extracellular and intracytoplasmic lumina.

Synchronous PitNETs/adenomas and TPPIs have also been described. Such associations accounted for 0.02% of cases collected in the German Pituitary Registry [71] with adrenocorticotropic hormone (ACTH)‐secreting corticotropinomas as the most common occurrence; TPPIs coexisting with immunonegative and gonadotroph tumors are also described [72, 73, 75, 76, 77].

## 12 MOLECULAR FEATURES

The molecular profile of TPPIs has been investigated in studies reporting a broad range of somatic mutations [5, 47, 78, 79, 80], and chromosome 1 imbalances [78]. Response to mitogen‐activated protein kinase (MAPK)/ERK inhibitors has been documented in a BRAFV600E mutant SCO [81, 82]. Krokker et al. identified miRNA and mRNA signatures distinctive of SCO compared to the normal posterior pituitary and of primary versus recurrent SCOs [83]. They also observed lower expression of DROSHA mRNA and protein, a nuclear class 2 ribonuclease that is essential to regulate the initiation of miRNA processing. Interestingly, 40% of downregulated miRNAs are mapped on chromosome 14q32, where NKX2.1 encoding TTF‐1 is located. Target prediction of miRNA and Gene Ontology analysis highlighted cell metabolism and cell cycle as the most dysregulated pathways, respectively. Mitochondrial Aconitase 2 was the most upregulated transcript, suggesting a pathogenetic role for this enzyme, which catalyzes the interconversion of citrate to isocitrate in the second step of the tricarboxylic acid cycle. A single case of GCT showed somatic missense mutations in SETD2 and PAX8 with variant allele frequency and amplification of chromosome 12 [84].

A recent, large multicenter study [8] investigated the pathological genetic and epigenetic profiles of 47 primary and recurrent TPPIs using DNA methylation analysis, copy number analysis, and targeted next‐generation sequencing. Methylation profiles were also generated from seven specimens of normal infundibulum and six specimens of posterior pituitary obtained from surgical biopsies or autopsies. Molecular profile was then correlated with patients' outcomes. With a median follow‐up of 5.1 years, no tumor related deaths were recorded.

Recurrent pathogenetic mutations in the MAPK/PI3K pathway, including *BRAF*, *FGFR1*, and *HRAS*, along with mutations in epigenetic regulators were enriched in SCOs and PTCs. No in‐frame fusions, focal amplifications, or deep deletions were observed. Some mutations were found at high allele frequency, indicating that they occurred early during tumorigenesis, while others were subclonal, suggesting they were acquired later during tumor development. No common genetic drivers across the TPPI spectrum were found to propose a unified molecular classification.

**FIGURE 4:**
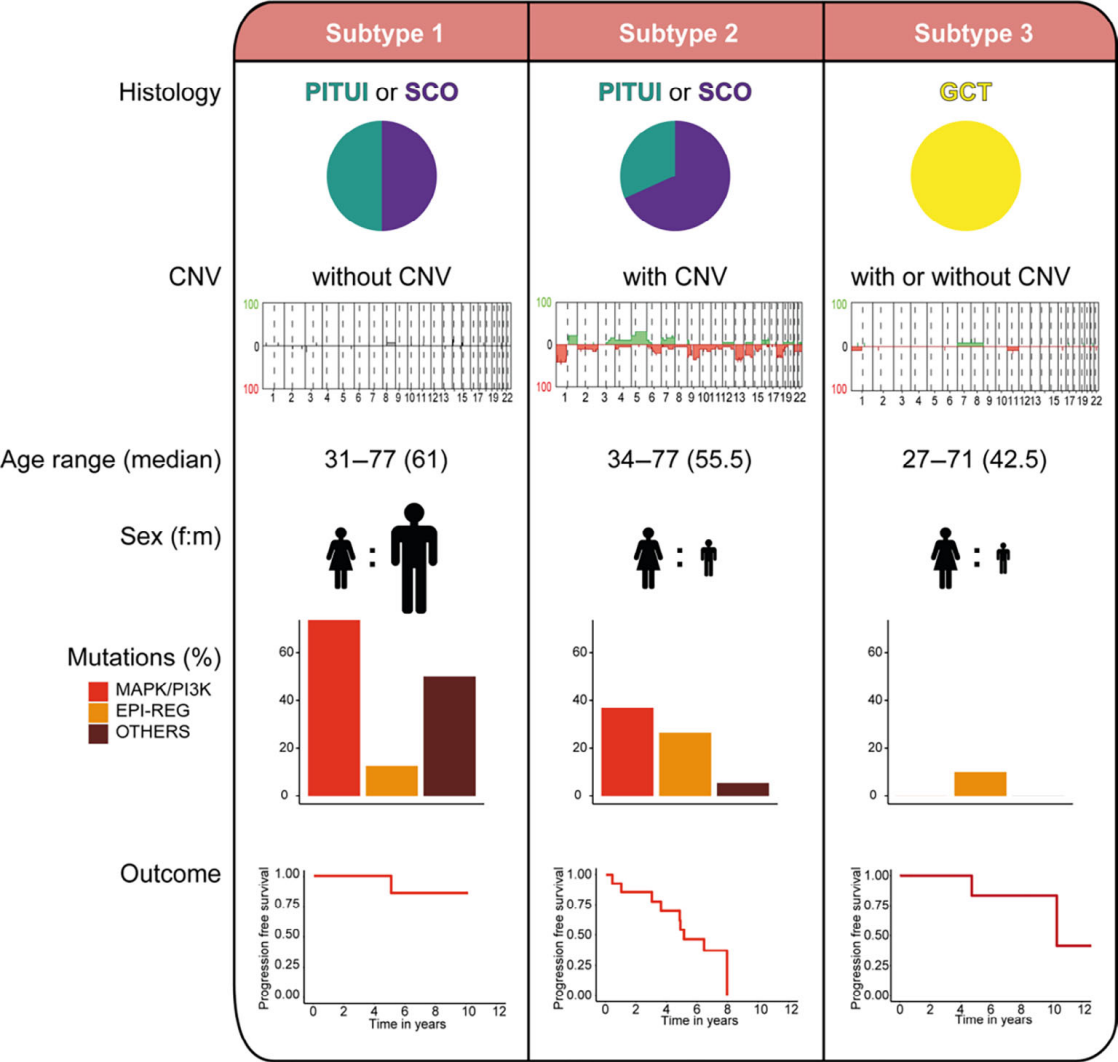
Synoptic representation of microscopic features, genetic profiles, and outcomes of the three groups of thyroid transcription factor‐1 (TTF‐1)‐expressing tumors of the posterior pituitary and infundibulum (reproduced from Schmid et al. [8]). CNV, copy number variation; GCT, granular cell tumor; SCO, spindle cell oncocytoma.

DNA methylation profiling stratified TPPIs in two large groups, one smaller group of lesions, and outliers. The largest group included MAPK/PI3K altered and epigenetic regulator altered tumors; the second largest group included GCTs and SCOs lacking recurrent mutations. Four SCOs, two of which included unusual light microscopic features, showed high CNVs and mutations in epigenetic regulators but lacked MAPK alterations (Figure 4).

CNV appeared similar when analyzed in separate areas of the same tumor and remained stable in recurrent lesions. No CNV were found in GCTs or in MAPK/PI3K‐altered tumors. The group with high chromosomal imbalances had the shortest progression free survival, with SCO displaying the highest relapse rate, while mutational status did not associate with worse outcomes.

## 13 PRIMARY PAPILLARY EPITHELIAL TUMOR OF THE SELLA

**FIGURE 5:**
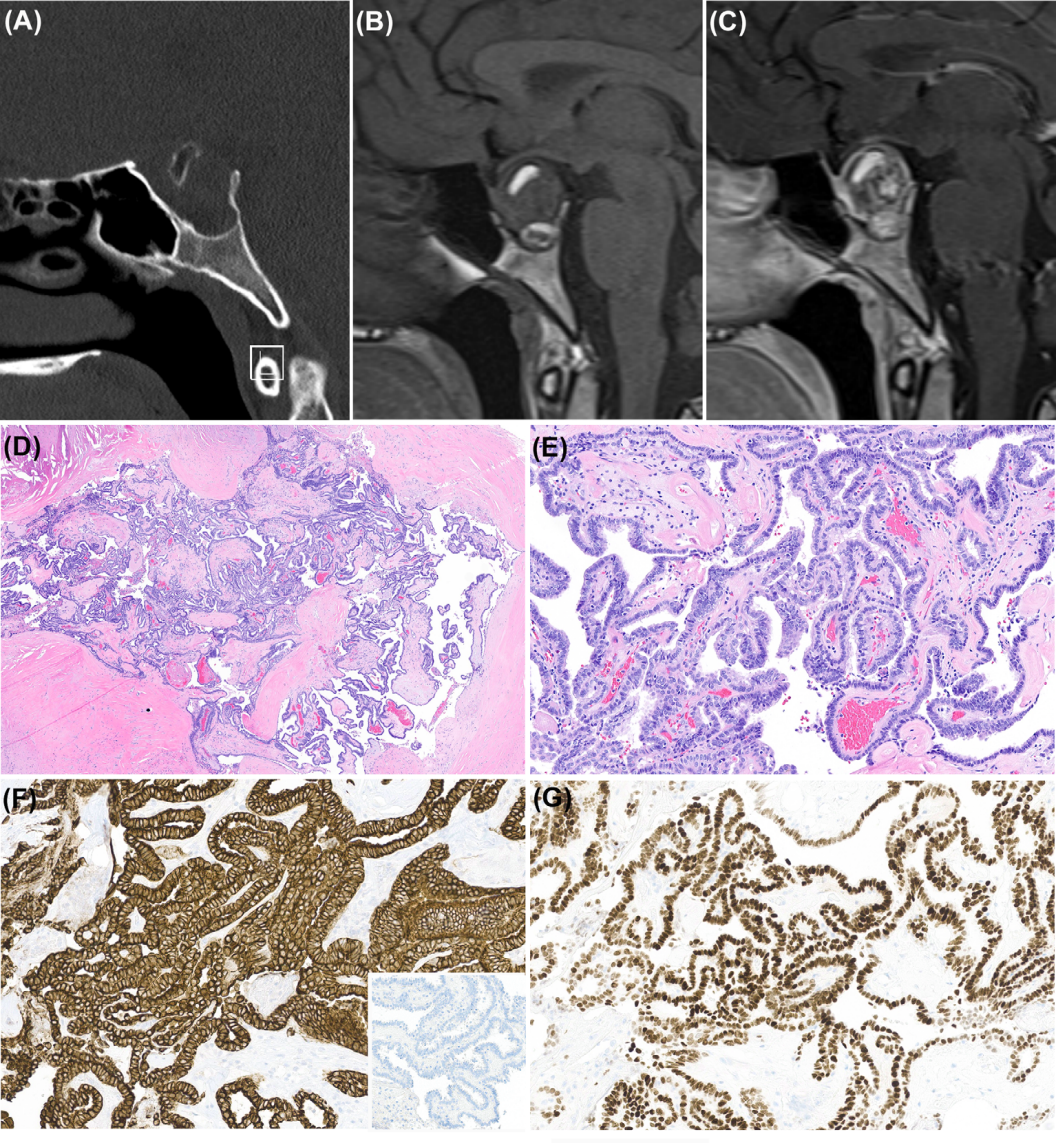
Example of primary papillary epithelial tumor of the sella; the bone window of the computed tomography scan shows expansion of the sella without erosion of sellar floor (A—sagittal reconstruction); sagittal T1‐weighted pre‐contrast (B) and post‐contrast (C) sequences show a sellar and suprasellar, solid and cystic lesion with inhomogeneous intensity and focal contrast enhancement. The tumor shows a striking papillary architecture (D—hematoxylin and eosin [H&E], ×4) with papillae lined by a single layer of unform cuboidal cells with basal nucleus (E—H&E, ×20). Neoplastic cells stain for cytokeratin 7 (F—immunoperoxidase, ×20) and TTF‐1 (G—immunoperoxidase, ×20). The inset of figure F shows the negative cytokeratin 20 (F inset—immunoperoxidase, ×20).

In 2020, Roncaroli et al. reported four patients with a sellar/suprasellar tumor showing a striking papillary architecture and a phenotype characterized by cytokeratin and TTF‐1 expression and proposed the descriptive term PPETS [85]. The series included three women (age 20, 26, and 42 years) and one 49‐year‐old man. The patients presented with varying signs and symptoms, including headache, amenorrhea, and visual symptoms, and were found to have solid and cystic sellar/suprasellar lesions ranging in size from 2.6 to 4.5 cm, encroaching in some cases on the chiasm and third ventricle, but without invasion of the cavernous sinus (Table 1). All tumors showed well‐formed papillae with fibrovascular cores lined by a single layer of tightly packed cuboidal or cylindrical cells with round to ovoid nuclei and eosinophilic cytoplasm. There was no mitotic activity. Some of the cases showed focal lympho‐plasmacytic infiltrates, clusters of macrophages, focal hemosiderin deposits, microcalcifications, or necrosis. The immunophenotype was unique compared to other sellar tumors with cytokeratin 7 expression, focal AE1/AE3 and CAM5.2, apical EMA expression, and intense and diffuse TTF‐1 expression, while stains for synaptophysin, chromogranin, and pituitary hormones, CK20, S100, GFAP, thyroglobulin, PAX8, napsin, and germ cell markers were negative. A limited next‐generation sequencing panel performed on three cases did not reveal any pathogenic variants. Based on their morphological features and immunophenotype, these tumors appeared to be distinct from the remaining TTF‐1‐positive sellar lesions previously discussed. They represented less than 0.1% of sellar tumors. The authors suggested that PPETS could represent the sellar/intracranial equivalent of low‐grade nasopharyngeal papillary adenocarcinoma, a slow‐growing tumor, typically cured by surgical excision, also characterized by a similar morphology and immunophenotype, showing diffuse TTF‐1 positivity. They also raised the possibility that rare tumors previously reported as ectopic choroid plexus papilloma of the sella and never tested for TTF‐1, may represent the same entity. A tumor likely representing part of the spectrum of PPETS, with prominent oncocytic changes, was discussed at the Diagnostic Slides Session of the American Association of Neuropathologist annual meeting in 2019 (DSS‐2019‐Case‐04 [pitt.edu] and DSS‐2019‐04.svs—DSS viewer [pitt.edu]). A recent example of this tumor we studied is illustrated in Figure 5.

**TABLE 1:** Summary of clinical features of the 11 published cases of primary papillary epithelial tumor of the sella.

References	Case	Age (years)	Sex	Location	Clinical findings	Pituitary hormones	GTR	Adjuvant therapy	Recurrence	Follow‐up (months)
Roncaroli et al. [85]	1	20	F	Sellar/suprasellar	Headache (3 mo), secondary amenorrhea (5 mo)	Slightly increased PRL (50.2 ng/mL), normo‐gonadotropic hypogonadism	Y	N	Y (at 10 mo)	Disease free (at 51 mo)
	2	26	F	Sellar/suprasellar	Recent amenorrhea and galactorrhea	PRL levels 52.5 ng/dL (0 min) and 50.05 (120 min), central hypoadrenalism and hypothyroidism, normo‐gonadotropic hypogonadism	Y	N	N	12
	3	49	M	Sellar/suprasellar	Visual symptoms	NA	Y	N	N	132
	4	42	F	Sellar/suprasellar	Trigeminal nerve, 3rd branch numbness	Normal	Y	N	NA	‐
Chen et al. [86]	5	59	M	Sellar	Dizziness, worsening headaches	Panhypopituitarism	NA	NA	NA	‐
Feng et al. [87]	6	29	F	Sellar	Blurred binocular vision bitemporal hemianopsia	Slightly decreased TSH	Y	N	N	28
	7	52	F	Sellar	Sudden headache, nausea, and vomiting	Slightly decreased thryrotropin, ACTH, and LH and increased PRL	Y	N	N	30
	8	49	F	Sellar	Blurred binocular vision	Normal	Y	N	N	120
	9	66	M	Sellar	Headache	Normal	Y	N	N	108
	10	31	F	Sellar	Amenorrhea and intermittent galactorrhea (2 years)	Slightly increased PRL	Y	N	N	123
Rima et al. [88]	11	44	F	Sellar/suprasellar	Progressive vision loss	Not available	Near total	NA	NA	‐

Abbreviations: ACTH, adrenocorticotropic hormone; F, female; LH, Luteinzing Hormone; M, male; mo, months; N, no; NA, not available; PRL, prolactin; TSH, Thyroid Stimulating Hormone; Y, yes.

In late 2022, Chen et al. have also reported a PPETS case in a 59‐year‐old man with typical clinical, imaging, morphological, and immunohistochemical features [86]. In this case, genome‐wide methylation profiling was performed using the Tumor Classifier software pipeline developed by the Heidelberg group [89, 90]. The methylation profile analysis did not match any of the previously characterized methylation classes, including choroid plexus tumors, PitNET/adenoma, and craniopharyngioma. While TTF‐1‐positive TPPIs including GCT, PTC, and SCO have a distinctive methylation profile [89], the tumor methylation profile confidence score for PTC was 0.3 and therefore not indicative of pituicyte origin. The authors suggested that PPETS may truly represent a novel tumor entity. No t‐distributed Stocastic Neighbor Embedding (t‐SNE) or Uniform manifold approximation and projection data was provided regarding the case.

Feng et al. [87] performed whole genome methylation analysis in three (of five) well documented PPETS and compared them to six GCTs, four PTC, and five SCO, seven central neurocytomas (which also express TTF‐1), seven choroid plexus papillomas, and seven control brains. Both unsupervised hierarchical clustering analysis and t‐SNE analysis showed the PPETS form a distinct cluster together with the TPPIs, separate from central neurocytoma and choroid plexus papilloma. The t‐SNE analysis of their three PPETS formed clustered, with the 29 TPPIs of the Heidelberg reference cohort [89] and with the large TPPI dataset published by Schmid et al. [8] (figure S2 of their paper). These findings and the similarity of CNV observed between the PPETs and the TPPIs suggested that they could still be part of the spectrum of TTF‐1‐positive TPPIs despite the differences in morphology and immunophenotype. A further case has very recently been described by Rima et al. [88].

Metastatic thyroid papillary carcinoma represents the main differential diagnosis of PPETS. In this respect, epigenetic profiling can help differentiate between the two lesions.

## 14 PRIMARY SELLAR ATYPICAL TERATOID RHABDOID TUMOR

Atypical teratoid /rhabdoid tumor is a rare embryonal tumor composed of poorly differentiated cells with a potential for divergent differentiation, along neuroepithelial, epithelial, and mesenchymal lineages [2]. It corresponds to a central nervous system WHO grade 4. The tumor is characterized by biallelic inactivation of *SMARCB1*, or rarely of *SMARCA4*. While it is typically a tumor of young children with a preferential involvement for the posterior fossa, a subset can occur in adults and involve the sella.

After a few case reports [3, 74, 91, 92, 93], the first series of sAT/RT was reported in 2017 by Nakata et al. [94], followed by Paolini et al. in 2018 [95] for a total number of 23 patients. All but one were adult females with a mean age of 43.8 (median 46; range 20–69). On imaging, all tumors involved the sella, some with invasion of the cavernous sinus and/or suprasellar extension; they often showed heterogeneous enhancement. Preoperatively, they were interpreted as nonfunctioning and/or aggressive PitNET/adenomas. In the majority of the patients for which clinical information was reported, surgical intervention only achieved subtotal tumor removal, prompting aggressive postoperative multimodal treatment including radiation and chemotherapy. The prognosis is poor; 13 of the 23 patients succumbed to disease progression. Median survival of patients with sAT/RT was reported as 30 months with a 1‐year survival estimate of 76.7%, significantly longer than median overall survival in children (11.1 months).

Histologically, sAT/RTs are composed of a dense proliferation of cells with a small to medium‐sized nucleus with vesicular chromatin and prominent nucleoli. Cytoplasm is variable, generally scant, with an eosinophilic, pale, or clear appearance. Rhabdoid cells are generally scarce. Mitotic figures are frequent and there is often evidence of necrosis. The histopathological features are those of a malignant tumor and appear to be relatively nonspecific, prompting a wide differential diagnosis.

AT/RT has a polyphenotypic immunoprofile with frequent expression, at least focally, of EMA, cytokeratin (CAM5.2), GFAP, vimentin, smooth muscle actin, and others. Such polyphenotypic profile can be very confusing if sAT/RT is not considered, leading to extensive immunohistochemical stains including multiple cell lineage markers to exclude other sellar high‐grade primary tumors or a metastasis. For instance, SALL4 expression may erroneously induce considering a germ cell tumor [96]. OCT3/4 and PLAP are, however, typically negative. A single immunostain highlighting the loss of SMARCB1 (INI1) before requesting a broad panel is enough to point toward the correct diagnosis.

Mutation or deletion of the SMARCB1 locus at 22q11.2 is the genetic hallmark of sAT/RT, which typically occur in a remarkably very low mutational burden and chromosomal imbalances. SMARCB1 is a component of the mammalian SWI/SNF complex involved in chromatin remodeling, transcriptional regulation, and ultimately mediating cell differentiation and lineage specification. Nearly all tumors show biallelic alterations in SMARCB1 through a variable combination of mechanisms, including pathogenic mutations with concurrent copy neutral loss of heterozygosity resulting in the absence of a normal allele, pathogenic mutations affecting both alleles, or homozygous deletion of chromosome 22q encompassing SMARCB1. Epigenetic and gene expression profile stratify AT/RTs into three subtypes: AT/RT‐Sonic Hedgehog (SHH), AT/RT‐TYR, and AT/RT‐MYC, showing respectively overexpression of proteins in the SHH and NOTCH signaling pathways, upregulation of proteins in the melanosomal pathway, and expression of the MYC oncogene and HOXC cluster genes. sAT/RT typically display a DNA methylation profile matching the one of the AT/RT‐MYC subgroup [97].

**FIGURE 6:**
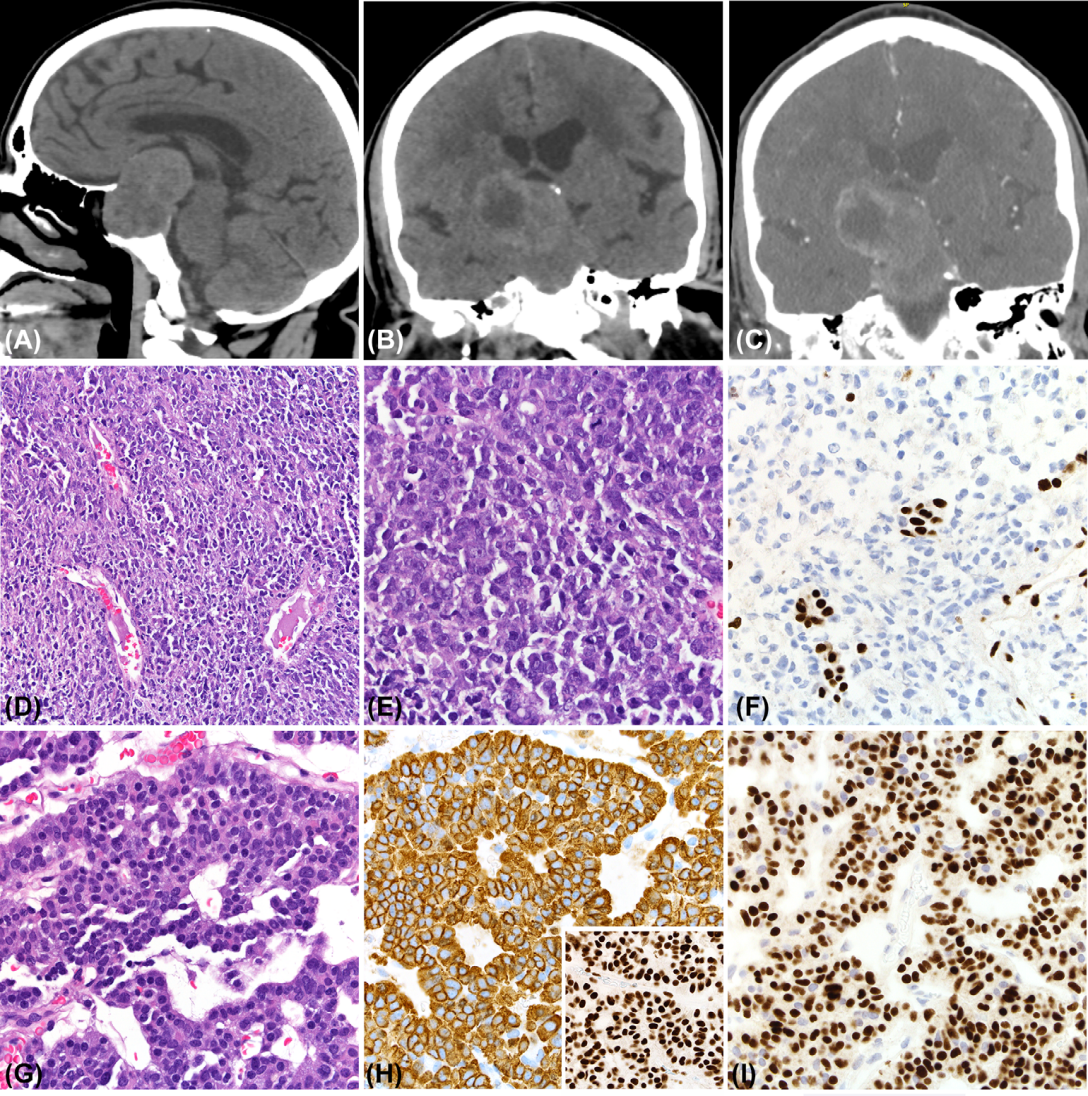
Sagittal and coronal reconstruction computed tomography scans show a large isodense and hypodense tumor eroding the sellar floor (A, B) with faint enhancement following contrast administration (C). Histologically, the lesion shows two distinct components; one is characterized by densely cellular sheets of atypical cells with scant cytoplasm and hyperchromatic nucleus (D—hematoxylin and eosin [H&E], ×4; E—H&E, ×20). INI‐1 is lost in tumor cells and retained in endothelial cells (F—immunoperoxidase, ×20); the residual gonadotroph tumor is composed of trabecular of uniform, mildly atypical cells (G—H&E, ×20) that express chromogranin A (H—immunoperoxidase, ×20), INI‐1 (H inset—immunoperoxidase, ×20), and SF‐1 (I—immunoperoxidase, ×20). No areas documenting the interface between sAT/RT and gonadotroph tumour were present in the specimen submitted to pathology.

Rare examples of sAT/RT including referrals to one author (CG; unpublished data) (Figure 6) have developed in patients with PitNET/adenoma, at times following radiotherapy [98]. The relationship between the original endocrine tumour and the sAT/RT, and the possible role of radiation remains to be determined.

In a series of primary collision tumors of the sellar region, a single case of sAT/RT concurrent with an adamantinomatous craniopharyngioma has been reported in a 23‐year‐old man, presenting with a large intra‐ and suprasellar cystic mass and in which craniopharyngioma and sAT/RT were intertwined with each other. The sAT/RT component, composed primarily of spindle cells and rhabdoid cells with focal mucus degeneration of the stroma, showed diffuse positive stain for vimentin, variable stain for synaptophysin, CD99, and EMA, and loss of expression of SMARCB1. This patient, diagnosed with craniopharyngioma and sAT/RT, was alive 18 months following surgery [99].

## 15 CONCLUSION

The sella is the site of a broad spectrum of tumors with distinctive pathological and molecular features. When dealing with sellar tumors lacking expression for pituitary hormones and lineage‐restricted pituitary transcription factors, TPPIs with unusual features, PPETs, and AT/RT must be considered. Epigenetic profiling and mutation analysis can help refine the diagnosis, inform prognosis and guide treatment.

## AUTHOR CONTRIBUTION

FR and CG have equally contributed to the conception, writing and editing of the manuscript.

## CONFLICT OF INTEREST STATEMENT

The authors declare no conflicts of interest.

## Data Availability

Data sharing is not applicable to this article as no new data have been created or analyzed.
